# Novel ideas to further expand the applicability of rhythm analysis

**DOI:** 10.1002/ece3.8417

**Published:** 2021-12-06

**Authors:** Lara S. Burchardt, Elodie F. Briefer, Mirjam Knörnschild

**Affiliations:** ^1^ Museum für Naturkunde ‐ Leibniz Institute for Evolution and Biodiversity Science Berlin Germany; ^2^ Institute of Animal Behavior Freie Universität Berlin Berlin Germany; ^3^ Behavioural Ecology Group Section for Ecology & Evolution Department of Biology University of Copenhagen Copenhagen Ø Denmark; ^4^ Université Paris‐Saclay Université Paris‐Sud CNRS UMR 9197 Institut des Neurosciences Paris‐Saclay Orsay France; ^5^ Smithsonian Tropical Research Institute Balboa Ancon Panama

**Keywords:** Fourier analysis, goodness‐of‐fit value, interonset interval, recurrence plot, rhythm analysis, *ugof*, universal goodness‐of‐fit‐value

## Abstract

The temporal structure of animals’ acoustic signals can inform about context, urgency, species, individual identity, or geographical origin. We present three independent ideas to further expand the applicability of rhythm analysis for isochronous, that is, metronome‐like, rhythms. A description of a rhythm or beat needs to include a description of its goodness of fit, meaning how well the rhythm describes a sequence. Existing goodness‐of‐fit values are not comparable between methods and datasets. Furthermore, they are strongly correlated with certain parameters of the described sequence, for example, the number of elements in the sequence. We introduce a new universal goodness‐of‐fit value, *ugof*, comparable across methods and datasets, which illustrates how well a certain beat frequency in Hz describes the temporal structure of a sequence of elements. We then describe two additional approaches to adapt already existing methods to analyze the rhythm of acoustic sequences of animals. The new additions, a slightly modified way to use the already established Fourier analysis and concrete examples on how to use the visualization with recurrence plots, enable the analysis of more variable data, while giving more details than previously proposed measures. New methods are tested on 6 datasets including the very complex flight songs of male skylarks. The *ugof* is the first goodness‐of‐fit value capable of giving the information per element, instead of only per sequence. Advantages and possible interpretations of the new approaches are discussed. The new methods enable the analysis of more variable and complex communication signals. They give indications on which levels and structures to analyze and enable to track changes and differences in individuals or populations, for instance, during ontogeny or across regions. Especially, the *ugof* is not restricted to the analysis of acoustic signals but could for example also be applied on heartbeat measurements. Taken together, the *ugof* and proposed method additions greatly broaden the scope of rhythm analysis methods.

## Graphical abstract

The temporal structure of animals’ acoustic signals can inform about many things. The description of these structures, namely, the rhythms, is only as good as their goodness‐of‐fit value indicates. We introduce a new universal goodness‐of‐fit value (*ugof*) comparable across methods and datasets and two other method additions to rhythm analysis. The new methods enable the analysis of more complex signals, and especially, the *ugof* is not restricted to the analysis of acoustic signals but could for example also be applied on heart‐beat measurements and greatly broadens the scope of rhythm analysis methods.

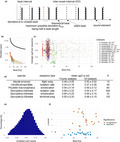

## 1 INTRODUCTION

In recent years, the temporal structure or rhythm of animal's acoustic signals has received increasing attention. Much emphasis lays on the development of methods to assess and quantify underlying temporal patterns (Burchardt & Knörnschild, 2020; Burchardt et al., 2019; Norton & Scharff, 2016; Ravignani & Norton, 2017; Saar & Mitra, 2008). The rhythm of an isochronous—that is, metronome‐like—element sequence is termed a “beat frequency” and is given in Hz. So far, three methods have been proposed in the context of bioacoustics for extracting exact beat frequencies in order to describe an isochronous element sequence: (1) Fourier analysis, which decomposes a signal into its sinusoidal components (Burchardt & Knörnschild, 2020; Saar & Mitra, 2008); (2) generate‐and‐test approach (GAT), where a series of acoustic signals are overlaid with an artificial beat to test which artificial beat frequencies resemble the series best (Norton & Scharff, 2016; Ravignani & Norton, 2017); and (3) interonset interval analysis (IOI), which allows the calculation of beat frequencies by averaging IOIs and transforming this rate into a frequency (Burchardt & Knörnschild, 2020).

Until now, studies on temporal structure or rhythm of animal's acoustic signals have often focused on quite simple sequences with an underlying isochronous structure (e.g., only one element type, visually uniform temporal structures, or short sequences; Burchardt & Knörnschild, 2020; Ravignani, 2018). Such a structure resembles a metronome sound, with constant beat and gap lengths. The above‐mentioned methods, GAT and Fourier analysis, together with the commonly used calculation of rates or frequency‐transformed rates (in Hz as in beats per second) describe these isochronous sequences well. However, for sequences containing various element types, subunits, and a strong variability between element duration and/or gap durations, such as skylark song (Briefer et al., 2010), nightingale song (Hultsch & Todt, 1981), whale song (Payne & McVay, 1971), or bat song (Behr & Helversen, 2004), the interpretation of results of exact beat frequency calculations described above becomes more difficult. Arising problems include the fact that all methods always give a “best‐fitting” beat frequency also in the case, that an isochronous beat is not suitable to describe the sequence, and this beat frequency can therefore be very misleading. Also, interpretation of results is very clear for small coefficient values (i.e., nPVI or coefficient of variation analysis, where low values are explicitly indicating low variability; Burchardt & Knörnschild, 2020; Cameron et al., 2019; Ravignani & Norton, 2017), but higher values are not as easily interpreted, as they could indicate a different rhythmic pattern than isochrony or indeed a random succession of elements (Burchardt & Knörnschild, 2020). Analyses of the rhythm of such vocalizations require the refinement of established methods or the development of new ones, in order to allow a description of sequences in a meaningful and comparable way between species.

Current problems related to existing methods are twofold. The first issue, which is independent of the complexity of the structure, is the limitations with which so‐called goodness‐of‐fit values quantifying how well a certain beat frequency describes an element sequence can be compared between species as well as between methods. These values exist for all three above‐mentioned methods to extract exact, best‐fitting beats (Burchardt & Knörnschild, 2020), but they inflict three problems: (a) They show complex correlations to, among other parameters, the number of elements in a sequence; (b) values differ depending on the method used, which precludes any comparison between studies using different methods; and (c) only one value can be obtained for the whole sequence that is being analyzed, without any information at the element level. The second issue that becomes important regarding the analysis of more complex sequences is that, so far, existing methods provide only one best‐fitting beat frequency when, in fact, the sequence might be best described by more than one beat frequency. Directly related, it might be interesting to look for subpatterns and analyze different parts of a sequence separately, to be able to depict rhythm changes within a complex sequence. The next challenge thus becomes to know where or what these subpatterns might be.

In this study, we propose three new ideas on how to extend the existing analyses options, as well as how to bypass certain limitations. First and foremost, we introduce a new universal goodness‐of‐fit value. Second, we suggest that reporting the 10 most prominent beat frequencies in a sequence instead of only the best‐fitting beat frequency in Fourier analysis, which implies the assumption that one beat frequency is enough, is essential to describe a complex temporal structure. Third, we encourage the use of recurrence plots to identify the substructures and subunits that could be of interest for further analysis. The reporting on the *ugof* is emphasized throughout the manuscript, as it is a true innovation for the field, while the two other methods are additions to already established analyses.

## 2 MATERIALS AND METHODS

Analyses were conducted on a total of six datasets, five of which were merely re‐analyzed for this study. These five datasets re‐analyzed for the *ugof* were datasets where beat frequencies (in Hz) had already been calculated (Burchardt & Knörnschild, 2020; Burchardt et al., 2019), and a fairly simple temporal structure could be inferred; three different acoustic signals of the Neotropical bat *Saccopteryx bilineata*: (1) 500 multisyllabic isolation call sequences (Knörnschild et al., 2012), (2) 142 multisyllabic territorial songs (Behr et al., 2006), and (3) 33 echolocation call sequences (Knörnschild et al., 2012); as well as (4) 49 isolation call sequences of the Neotropical bat *Carollia perspicillata* (Knörnschild et al., 2013) and (5) 60 echolocation sequences of *Physeter macrocephalus* (Bøttcher et al., 2018; Tønnesen et al., 2018). All raw datasets were acoustic recordings in which the starts of elements were labeled manually or automatically depending on the dataset using the oscillogram. Timepoints were then extracted and used for further analysis.

The same procedure was used on a sixth dataset. We newly performed a rhythm analysis on a dataset of flight songs of the skylark, *Alauda arvensis* (for details on recordings, see Briefer et al., 2008, 2010). The song produced by males of this species during the breeding season while in flight is very complex: Each individual can combine more than 300 different syllables in its song, giving rise to a lot of variation (Aubin, 1982; Briefer, Aubin, et al., 2008; Briefer, Rybak, et al., 2008). The use of existing methods on such song, namely, reporting only the one best‐fitting beat frequency per sequence as calculated in Fourier analysis and the resulting goodness‐of‐fit values, proved to be insufficient for describing the rhythmic structure of this system. We therefore developed a goodness‐of‐fit value and re‐evaluated how to best report results of the Fourier analysis, to further facilitate comparability between methods and species, for example, through enabling the description of both simple and more complex patterns with the same methods, but also by making the various existing methods themselves more comparable. We introduce a newly established universal goodness‐of‐fit value, and we discuss additions to existing methods (Fourier analysis and recurrence plots), with the overall purpose to further advance rhythm analysis, its applicability, and comparability.

Our universal goodness‐of‐fit value is tested both on the skylark dataset and the five already published datasets. For the re‐evaluation of Fourier analysis results and recurrence plots, we focus on the complex dataset of skylark flight songs.

### 2.1 Introducing a universal goodness‐of‐fit value

**FIGURE 1:**
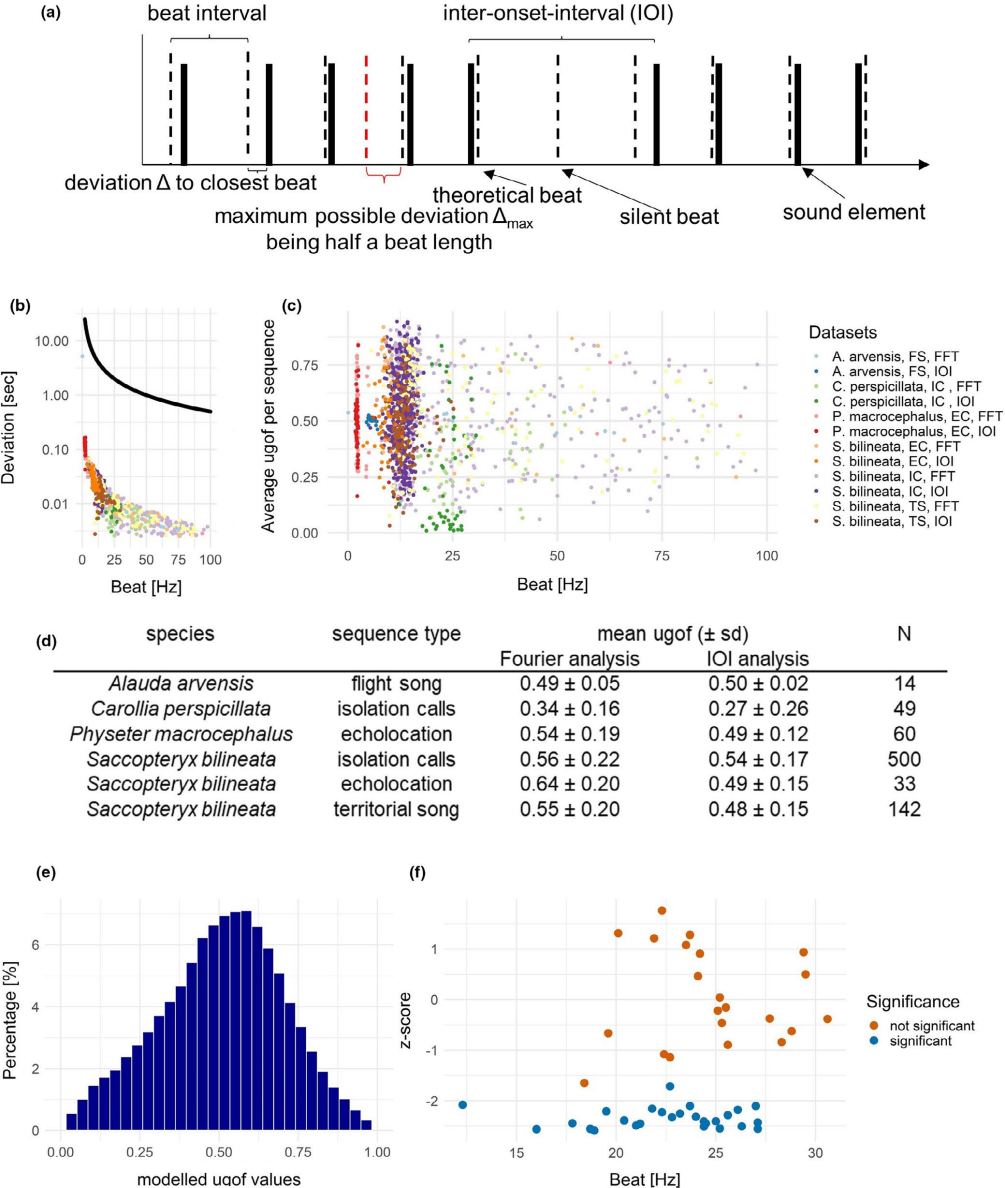
(a) Theoretical element series (solid black elements) with an overlaid beat (dashed lines) of a certain beat frequency in Hertz. The maximum possible deviation for any element is half the beat duration (Δ_max_). It is set in relation to the absolute deviation of an element to its closest beat (Δ). Other important concepts visualized are as follows: interonset intervals and silent beats. (b) The theoretical maximum deviation per beat (in black) and actual deviations (as mean per sequence) measured from six datasets and for two calculated beat frequencies each. Both deviations change in the same way depending on the corresponding frequency, and actual deviations are much smaller than maximum possible deviations. (c) *ugof* calculated for best‐fitting beat frequencies based on Fourier analysis and IOI analysis for six datasets. No correlation can be seen between *ugof* and beat frequency. (d) Tabular comparison of mean *ugof* per dataset for both beat calculation methods. Fourier analysis yields better results (lower *ugof*) *only* for the complex skylark song. (e) Distribution of *ugof* calculated for beat frequencies from 0.1 to 100 in 0.01 Hz increments for all sequences of *Carollia perspicillata* isolation call sequences, to be able to calculate *z*‐scores. (f) *z*‐scores as calculated based on the modeled *ugof* for beat frequencies of 49 isolation call sequences of *C*. *perspicillata* using IOI analysis. Significant values are in blue, and nonsignificant values in orange. The differences between significant and not significant beat frequencies could correlate with different individuals and potentially be connected to the relevance of beat production as a fitness indicator. EC, echolocation calls; FFT, beat frequencies calculated with a fast Fourier transformation (Fourier Analysis); FS, flight song; IC, isolation call sequences; IOI, beat frequencies calculated with IOI analysis; TS, territorial song

We propose a new, universal goodness‐of‐fit value that can be applied to any possible description of a temporal structure relying on frequencies; we term it *ugof* for “universal goodness‐of‐fit value.” It is a value that is calculated for every element in a sequence and can then be summarized for a whole sequence or any other desired grouping (e.g., individuum, group, sequence type). A theoretical beat describes a sequence well when there are only small deviations between the original elements and the theoretical beats of the best‐fitting beat frequency. One element always lies between two theoretical beats. Therefore, the maximum deviation possible equals to half of the theoretical beat length since one will always search for the deviation to the next closest beat (Figure 1a). We can thus describe a particular deviation as the ratio between the actual deviation to the next theoretical beat and the maximum deviation for the calculated best‐fitting beat (Equation 1).

(1) $$ugof=\frac{\left|\Delta \right|}{\Delta_{\text{max}}},$$

where *ugof* is the universal goodness‐of‐fit value, |Δ| is the absolute deviation to closest theoretical beat, and Δ_max_ is the maximum possible deviation (half a beat duration).

Both parameters, the maximum possible deviation to the next beat (Δ_max_) and the actual deviation (|Δ|), need to change in the same way depending on the corresponding frequency for the method to be universally applicable. This is depicted in Figure 1b, showing the theoretical maximum possible deviations (Δ_max_) for beats of beat frequencies of up to 100 Hz (in black) and the actual deviations (|Δ|) which we measured for a total of 804 sequences and of two beat frequencies for each sequence (calculated with two different methods: one with Fourier analysis, i.e., 12.1 Hz, and the other one with the IOI approach, i.e., 13.4 Hz), resulting in 1608 datapoints (in color). The actual deviations are indeed much lower than the maximum possible deviations (Figure 1b). By dividing the actual deviation (|Δ|) by the maximum possible deviation (Δ_max_) as shown in the equation above, we get the *ugof* as a ratio that can easily be transformed into a percentage (by multiplying with 100) if required. The smaller *ugof* is, the closer the original elements of a sequence are to the theoretical beats. The resulting value (*ugof*) is independent of the number of elements in the sequence, the sampling length, or the number of silent beats in a sequence (Figure 1a). It is also independent of the best‐fitting beat frequency it is describing (Figure 1c).

We calculated the *ugof* of six datasets and for two different beat frequencies per sequence, that is, using two different methods: The first beat frequency we calculated the *ugof* for was based on the Fourier analysis and the second best‐fitting beat frequency was based on the IOI approach (Figure 1c,d, as calculated with Fourier analysis and IOI analysis in previous studies; Burchardt & Knörnschild, 2020; Burchardt et al., 2019). To be able to evaluate and interpret a single *ugof*, we modeled the distribution of *ugofs* for a dataset. To this aim, we calculated *ugof* from 0.1 to 100 Hz in 0.01 Hz increments for all element sequences in the dataset. To illustrate what we mean by this, let us assume we have a sequence A. For this sequence A, for which we know when each element in the sequence starts, we calculate *ugof* for 1000 beat frequencies (0.1–100 Hz in 00.1 Hz increments), by calculating the actual deviations (|Δ|) as well as the maximum possible deviations (Δ_max_) for each frequency. Figure 1e shows the results of these calculations for a dataset of 49 isolation call sequences of the bat *Carollia perspicillata* (9991 *ugof* values for 49 sequences, giving us a distribution of 489,559 values). We then used the mean and standard deviation of this Gaussian distribution to evaluate any single best‐fitting beat frequency as calculated with the IOI approach. We can calculate *z*‐scores for every *ugof* by subtracting the mean of the distribution of *ugof* values for the given dataset (i.e., isolation call sequences of *C*. *perspicillata)* from the *ugof* in question (i.e., the *ugof* as calculated for the best‐fitting beat frequency with the IOI approach) and dividing the difference by the standard deviation of the distribution. A calculated *z*‐score can than easily be matched to the corresponding *p*‐value using *z*‐score tables (i.e., Fisher & Yates, 1964; Rohatgi & Saleh, 2015). This allows us to investigate whether a calculated beat frequency fits the element sequence significantly better than what could be expected depending on the calculated distribution of *ugof* for a specific dataset. We only considered negative *z*‐scores as possibly significant, as a negative *z*‐score indicates that the corresponding value is *below* the distributions mean (Figure 1e). A positive *z*‐score would, on the other hand, indicate that the corresponding *ugof* is *above* the distribution mean, and could also be significant, but would then fit significantly worse than expected by the underlying distribution.

This approach of using *z*‐scores, and therefore the possibility to calculate *p*‐values for different production rhythms, is mainly useful for comparability reasons, in order to assess which animal or individual can better keep a stable (theoretical) beat and to eventually answer the question why that is. We do *not* want to propose that a sequence would only be well described by an isochronous beat that results in a significant *ugof*. To illustrate the methods, we calculated *z*‐scores for beat frequencies in the dataset at hand (isolation calls of *C*. *perspicillata*) based on the IOI approach, as these resulted in on average smaller *ugof* compared to beat frequencies calculated with Fourier analysis (Figure 1d). The values were calculated for beat frequencies with a resolution of two decimal points (i.e., 12.11 or 28.84 Hz). This analysis revealed that *ugof* can sometimes change strongly within small increments. We thus suggest that it is reasonable to have a look at *ugof* within 1 Hz of the detected best‐fitting beat frequency to be aware of the possible sensitivity of the method, especially when having to deal with a low‐frequency resolution when using the Fourier analysis. The frequency range in which to check the *ugof* should take the frequency resolution into account. For instance, if the frequency resolution of the Fourier analysis is very coarse (see Burchardt & Knörnschild, 2020, for discussion), a certain beat frequency might not be found by the Fourier analysis, but a *ugof* could nevertheless be calculated. To be aware of that issue and to be certain about the calculated exact beat frequencies, this approach is suggested. The resulting *z*‐scores for the best‐fitting beat frequencies of the IOI analysis for isolation calls of *C*. *perspicillata* are shown (Figure 1f). We found that *z*‐scores vary considerably within the dataset. This might illustrate differences in beat production abilities between individuals, as some individuals might produce sounds in a more consistent/rhythmic way than others, which, in turn, could constitute a fitness indicator. In addition, differences between significant and not significant production rhythms could be related to different situations, that is, different arousal/motivation or urgency levels. For further discussion on this please see the appendix, there the modeled *ugof* values and *z*‐scores for all datasets are shown (Figures A1–A6).

### 2.2 Additions to existing methods of rhythm analysis

#### 2.2.1 Ten highest peaks of Fourier analysis

**FIGURE 2:**
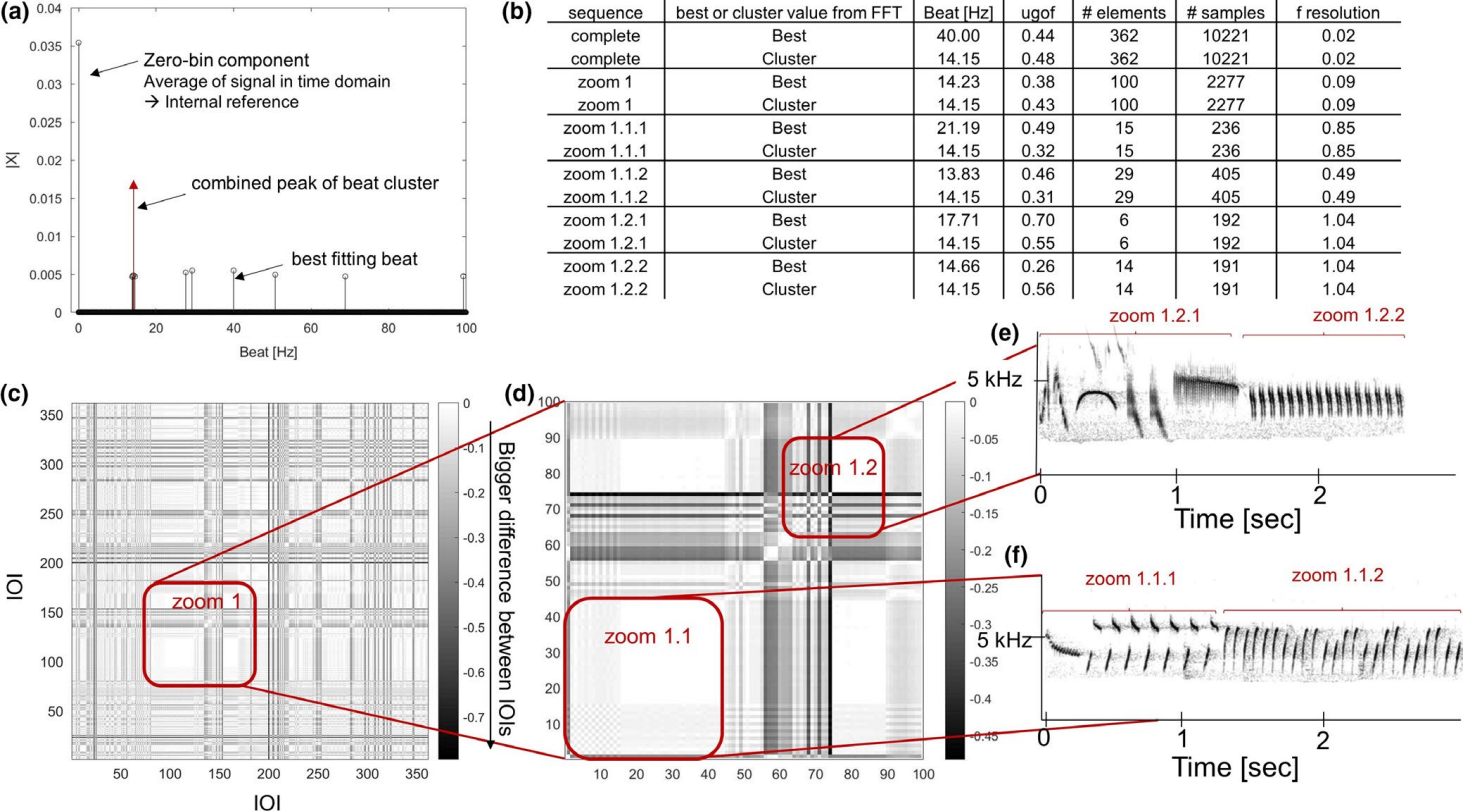
Exemplary results of the rhythm analysis of an excerpt from the complex flight song of the skylark *Alauda arvensis*. (a) Amplitude plot of Fourier analysis. Beat frequency is depicted on the *x*‐axis and the amplitude of the ten highest peaks—as calculated by a fast Fourier transformation—on the *y*‐axis. The highest peak is always the zero‐bin component at 0 Hz; it is the average of the signal in the time domain, where elements were encoded in a binary sequence (it is not relevant to find the best‐fitting beat, but a by‐product of the data transformation into a binary sequence and only shown for transparency and explanation). One very strong cluster can be identified; a summary of this cluster might depict the temporal structure better than the detected single highest peak. (b) The table reports relevant parameters of the rhythm analysis for the five units depicted in the figure. (c) Recurrence plots of the complete example sequence: All interonset interval (IOI) pairings in the sequence are compared to each other, forming a symmetric comparison of every IOI to every other IOI in the sequence. The Euclidean distance between any IOI pairing is color‐coded. More different pairs of IOIs are characterized by longer distances and darker colors. The corresponding audio is supplied. (d) Zoom into a section of 100 elements (11.2 s) of the song sequence. A very consistent series of IOIs can be observed at the beginning, followed by some slight changes, and in the end again, a very consistent pattern. (e) Spectrogram of the zoom 1.2 section, which can further be divided into a variable pattern (zoom 1.2.1) and a very consistent pattern (zoom 1.2.2). (f) Spectrogram of the zoom 1.1 section, which can further be divided into two consistent patterns (zoom 1.1.1 and 1.1.2)

Especially in more complex signals, such as bird song comprised of various motifs or phrases (Aubin, 1982; Hultsch & Todt, 1981; Kroodsma, 2005), it seems inappropriate to assume that one beat frequency could be enough to describe a sequence. The IOI approach seems particularly unsuitable here, as it simplifies the temporal structure (Burchardt & Knörnschild, 2020). The Fourier analysis, on the other hand, gives a very detailed picture of all beat frequencies that make up the sequence. It decomposes any signal into its sinusoidal components, which are nothing else but frequencies. A sequence of an animal's acoustic signal is transformed into a binary sequence, where an element onset is encoded as “1” and everything else encoded as “0.” A fast Fourier transformation is then conducted on this binary sequence (Ravignani & Norton, 2017; Saar & Mitra, 2008). In a recent publication, we settled to describe a sequence by the beat frequency that contributed the most to the description of a sequence, that is, the one that gave the highest amplitude in the Fourier analysis's frequency domain, and only reported this most prominent frequency (Burchardt & Knörnschild, 2020). We now propose, as an alternative, to report the ten most prominent frequencies. This would allow the detection of frequency “clusters” (red circles in Figure 2a). None of the frequencies in a cluster might have the highest peak. However, when combined (summed up), they surely describe a series better than a single, slightly higher peak. Therefore, it could also be an option to report a summary, average, or range of a particular cluster to describe a particular sequence. This gives a much more detailed result, which can be used as basis for decisions about how to proceed or what to report. We suggest looking at the ten highest peaks, as it is a reasonably high number to find possible clusters, without reporting beat frequencies that have only very small explanatory values for the sequence. Nevertheless, for certain sequences, it may be most informative to report only the five highest peaks or the twenty highest peaks. An alternative solution might be to consider reporting the number of peaks that explain a certain percentage of the sequence's rhythm. However, such percentage is not easily accessible and might lead to a very high number of peak (e.g., >100).

#### 2.2.2 Recurrence plots

The recurrence plot, originally used in chaos theory (Eckmann et al., 1987; Marwan, 2008), is an easy way to visualize the overall temporal structure of a sequence and to find subunits (Ravignani & Norton, 2017). It depicts the distance between any IOI pair in the sequence that is to be analyzed. Every possible IOI pair is compared, the Euclidean distance is measured and plotted (Burchardt & Knörnschild, 2020; Ravignani & Norton, 2017). Differences are color‐coded in the plots; the darker a comparison, the more different are the two compared IOIs. Subunits with very different temporal structures can be easily spotted in such a plot, namely, as a “break” in the pattern (Figure 2d). When analyzing new acoustic signals, where knowledge about functional units such as motifs is scarce, such temporal breaks could easily show where a new motif or phrase starts. Furthermore, different subunits might have different beat frequencies that convey meaning, but that cannot be resolved with an overall best‐fitting beat frequency. As shown in Figure 2b, identified subunits can then be analyzed to extract their specific best‐fitting beat frequency, in order to see whether they fit the overall temporal structure or not.

## 3 RESULTS

### 3.1 Results of an exemplary analysis of skylark flight song

To illustrate the proposed additions, we analyzed an excerpt from the complex flight song of a skylark. The specific sequence has a duration of 51.3 s and contains 362 elements (for details on recording, see Briefer, Rybak, et al., 2008, 2010). We calculated the best‐fitting beat frequency of the whole sequence and of five exemplary subunits (there are more in the entire sequence), which we identified via recurrence plots (Figure 2c–f). As can be seen in Figure 2a, there is a strong cluster of beat frequencies with high descriptive value for this sequence. To calculate the universal goodness of fit, we not only used the single best‐fitting beat frequency (indicated as “Best” in 2b), but also the beat frequency of the cluster mean (indicated as “Cluster” in 2b). The recurrence plot zoom 1.1 visualizes the subsequent switching between two element types followed by a series of very similar IOIs, which correspond to a single element type. In the recurrence plot zoom 1.2, on the other hand, we see more variability, looking at both the recurrence plot and the spectrogram in 2e; there we can see various element types, then a “break” (black line) in the recurrence plot indicating a high difference between the two adjacent IOIs, followed again by a very stereotyped subunit (zoom 1.2.2). The calculated *ugofs* gave some interesting insights. (1) The *ugof* for the best‐fitting beat frequency of the whole sequence was indeed higher than the one for the cluster‐beat frequency, but (2) the cluster‐beat frequency described some of the subunits well, sometimes even better than the best‐fitting beat frequency calculated for the subunits themselves. This was true especially for the four small subunits (zoom 1.1.1 and 1.1.2, and zoom 1.2.1 and 1.2.2). Only one out of four sequences here showed a better (i.e., smaller) *ugof* for the best‐fitting beat frequency (zoom 1.2.2) compared to the *ugof* calculated with the cluster‐beat frequency. However, here, both the cluster‐beat frequency and the best‐fitting beat frequency were very similar and, due to a lower frequency resolution in the Fourier analysis, the better fitting cluster‐beat frequency could mathematically not be found (see Burchardt & Knörnschild, 2020, for an explanation on frequency resolution in Fourier analysis). This is further proving the point that beat frequencies calculated by Fourier analysis with a low‐frequency resolution need to be handled and interpreted with care (Burchardt & Knörnschild, 2020).

## 4 DISCUSSION

Analyzing the temporal structure of animals’ acoustic signal is relevant for addressing many research questions, such as species discrimination, physiological correlates like couplings to wingbeat or respiration, mating preferences or arousal coding (Burchardt et al., 2019; David et al., 2003; Manser, 2001; McRae, 2020; Norton & Scharff, 2016). Other questions include duetting or the development of temporal structures during ontogeny (Pika et al., 2018; Sasahara et al., 2015; Yoshida & Okanoya, 2005). Many analyses conducted by bioacousticians include temporal parameters. We already indicated in an earlier paper (Burchardt & Knörnschild, 2020) that information, such as small scale interindividual differences, might be lost by focusing only on the commonly used “element rates,” mostly called “syllable rates” (Douglas et al., 2005; Manser, 2001; McRae, 2020). Using element rates or calculating beat frequencies per sequence by transforming the element rate into a frequency could be described as a “spyglass” approach, mostly useful for studying highly temporally consistent communication signals (i.e., echolocation of bats or whales). It is useful when investigating a species’ rhythm or other analyses that only require this level of detail. For more complex communication signals, or in cases when fine scale intra‐individual differences or fine scale differences between contexts might play a role, the “magnifying glass” approach of the Fourier analysis should be used instead. Our newly established *ugof* clearly supports this claim, as our analyses revealed better results (indicated by lower *ugofs*) when using the Fourier analysis compared to the IOI approach *only* for the very complex skylark flight song.

Our suggested additions to already established methods make these aims of not losing relevant and interesting information during the analysis of temporal parameters easier to reach. These new methods allow a comparison of rhythmicality both between studies and species, which was not easy beforehand. Analyzing recurrence plots to make an educated decision on which subpatterns to analyze can also be of interest when facing completely new acoustic signals. Clear temporal breaks, as can be seen in the recurrence plots shown above (Figure 2e), could easily indicate where a new motif or phrase starts. Distinguishing contexts or analyzing syntax could be backed up by such analyses of the underlying temporal structure (or vice versa). An example for this could be research on dialects. For instance, microgeographic differences between male skylarks’ flight song are mostly based on differences in the syllable and phrase repertoire composition (Briefer, Aubin, et al., 2008). Since such phrases show a distinguishable temporal patterning (i.e., higher tempo; Briefer, Aubin, et al., 2008), they could be automatically detected using these methods.

Our newly established universal goodness‐of‐fit value enables every researcher, whether reading such a study or conducting it, to grasp the rhythmicity of an individual, a single sequence, or a species, by looking at one number alone, which can be accompanied by a *p*‐value. A number between 0 and 1, with smaller numbers indicating a better fit, is easy to interpret. No understanding of correlations within the data is needed. Furthermore, it can be easily determined which of the methods used to describe a sequence (i.e., Fourier analysis or IOI approach) captures most of the underlying temporal structure, or whether a subunit has a beat frequency different from the beat frequency of the whole sequence. It is to be noted that a value of 1 is not expected, as this would mean that all elements of the sequence lie exactly between two beats, which would indicate that they all perfectly fit the theoretical beat, but phase displaced. Furthermore, it is the first proposed goodness‐of‐fit value that is calculated per element and not per sequence, therefore enabling bioacousticians to answer even more interesting questions about sublevel structures. Such questions could be about which elements “drive” a beat frequency or break it, which could then shed light on the accentuation of elements. It could furthermore be applied to the analysis of bivariate signals, for example, to quantify the synchronization between, for example, wingbeat and echolocation of flying bats (Kalko, 1994; Moss et al., 2006; Ratcliffe et al., 2011; Schnitzler, 1971) or flight song and wingbeat in the skylark. This could be done in two different ways: first, by calculating *ugofs* for the same rhythm for both signals and comparing them, or second, by using one of the signals—the potential driver of the temporal structure—as “theoretical beat,” to calculate how much the second signal differs from the oscillator. This approach could also be used for the analysis of duetting or synchronized animal sounds such as frog choruses.

On another note, these methods are not only useful when analyzing acoustic signals. They can be used on the temporal structure of anything, may it be a certain behavior or physiological processes such as wingbeat, heartbeat, or respiration. All processes of interest can easily be transformed in a way to enable the analysis; for example, instead of interpreting the result in Hz, which is one beat per second, we could interpret it as beats/occurrences per hour, day, or more abstract processes such as a reproductive cycle. We can subsequently calculate our GAT, IOI, or Fourier analysis on that particular time scale and retransform the results back to the original time scale. More concrete applications of such method could be to quantify movement errors in another way than qualitatively by a human observer, as was done recently in a study on precision of dance movements in professional and nonprofessional dancers (Karageorghis et al., 2019). It could also be used to quantify accuracy in tapping tasks, which are frequently used to assess the medical status of patients (Criswell et al., 2010; Roalf et al., 2018), in order to improve accuracy in the field (Roalf et al., 2018). Potentially, our method could even be used in competitive sports to assess movement structure and timing.

Rhythm analysis methods that have been developed for acoustic analysis could thus allow an even wider range of researchers in answering questions such as movement errors, sleep cycle analysis, finger tapping tasks, circadian rhythms, or various other research areas, possibly even in economics or engineering where temporal structures of processes are of utmost importance as well.

## CONFLICT OF INTEREST

The authors declare that the research was conducted in the absence of any commercial or financial relationships that could be construed as a potential conflict of interest.

## AUTHOR CONTRIBUTIONS

Lara Sophie Burchardt: Conceptualization (lead); Data curation (supporting); Formal analysis (lead); Funding acquisition (equal); Investigation (lead); Methodology (lead); Project administration (lead); Software (lead); Validation (lead); Visualization (lead); Writing – original draft (lead); Writing – review & editing (equal). Elodie Briefer: Data curation (lead); Funding acquisition (equal); Writing – review & editing (equal). Mirjam Knoernschild: Funding acquisition (equal); Project administration (supporting); Resources (lead); Supervision (lead); Writing – review & editing (equal).

## Supporting information

Supplementary Material

## Data Availability

The raw Skylark IOI data are available at the Museum für Naturkunde Berlin Data Repository at https://doi.org/10.7479/m0ge‐6a51. Codes for the rhythm analysis, including Fourier analysis and the *ugof*, are provided at: https://github.com/LSBurchardt.

## References

[ece38417-bib-0001] Aubin, T. (1982). Habituation au chant territorial chez l'alouette des champs (*Alauda arvensis* L.). Biology of Behaviour, 7, 353–362.

[ece38417-bib-0002] Behr, O. , & Helversen, O. (2004). Bat serenades – Complex courtship songs of the sac‐winged bat (*Saccopteryx bilineata*). Behavioural Ecology and Sociobiology, 56, 106–115.

[ece38417-bib-0003] Behr, O. , Helversen, O. , Heckel, G. , Nagy, M. , Voigt, C. C. , & Mayer, F. (2006). Territorial songs indicate male quality in the sac‐winged bat *Saccopteryx bilineata* (Chiroptera, Emballonuridae). Behavioural Ecology, 17, 810–817.

[ece38417-bib-0004] Bøttcher, A. , Gero, S. , Beedholm, K. , Whitehead, H. , & Madsen, P. T. (2018). Variability of the inter‐pulse interval in sperm whale clicks with implications for size estimation and individual identification. The Journal of the Acoustical Society of America, 144(1), 365–374. 30075661 10.1121/1.5047657

[ece38417-bib-0005] Briefer, E. , Aubin, T. , Lehongre, K. , & Rybak, F. (2008). How to identify dear enemies: The group signature in the complex song of the skylark *Alauda arvensis* . Journal of Experimental Biology, 211, 317–326. 18203986 10.1242/jeb.013359

[ece38417-bib-0006] Briefer, E. , Osiejuk, T. S. , Rybak, F. , & Aubin, T. (2010). Are bird song complexity and song sharing shaped by habitat structure? An information theory and statistical approach. Journal of Theoretical Biology, 262(1), 151–164. 19782691 10.1016/j.jtbi.2009.09.020

[ece38417-bib-0007] Briefer, E. , Rybak, F. , & Aubin, T. (2008). When to be a dear enemy: Flexible acoustic relationships of neighbouring skylarks, *Alauda arvensis* . Animal Behaviour, 76, 1319–1325.

[ece38417-bib-0008] Briefer, E. , Rybak, F. , & Aubin, T. (2010). Are unfamiliar neighbours considered to be dear‐enemies? PLoS One, 5(8), e12428. 10.1371/journal.pone.0012428 20865148 PMC2928747

[ece38417-bib-0009] Burchardt, L. S. , & Knörnschild, M. (2020). Comparison of methods for rhythm analysis of complex animals’ acoustic signals. PLOS Computational Biology, 16(4), e1007755. 32267836 10.1371/journal.pcbi.1007755 PMC7141653

[ece38417-bib-0010] Burchardt, L. S. , Norton, P. , Behr, O. , Scharff, C. , & Knörnschild, M. (2019). General isochronous rhythm in echolocation calls and social vocalizations of the bat *Saccopteryx bilineata* . Royal Society Open Science, 6, 181076. 30800360 10.1098/rsos.181076 PMC6366212

[ece38417-bib-0011] Cameron, D. J. , Zioga, I. , Lindsen, J. P. , Pearce, M. T. , Wiggins, G. A. , Potter, K. , & Bhattacharya, J. (2019). Neural entrainment is associated with subjective groove and complexity for performed but not mechanical musical rhythms. Experimental Brain Research, 237(8), 1981–1991. 31152188 10.1007/s00221-019-05557-4 PMC6647194

[ece38417-bib-0012] Criswell, S. , Sterling, C. , Swisher, L. , Evanoff, B. , & Racette, B. A. (2010). Sensitivity and specificity of the finger tapping task for the detection of psychogenic movement disorders. Parkinsonism & Related Disorders, 16(3), 197–201. 20005766 10.1016/j.parkreldis.2009.11.007 PMC2829355

[ece38417-bib-0013] David, J. A. D. O. , Zefa, E. , & Fontanetti, C. S. (2003). Cryptic species of *Gryllus* in the light of bioacoustic (Orthoptera: Gryllidae). Neotropical Entomology, 32, 75–80.

[ece38417-bib-0014] Douglas, L. A. , Dawson, S. M. , & Jaquet, N. (2005). Click rates and silences of sperm whales at Kaikoura, New Zealand. The Journal of the Acoustical Society of America, 118(1), 523–529. 16119371 10.1121/1.1937283

[ece38417-bib-0015] Eckmann, J.‐P. , Kamphorst, S. , Oliffson, S. , & Ruelle, D. (1987). Recurrence plots of dynamical systems. Europhysics Letters, 4(9), 973–977.

[ece38417-bib-0016] Fisher, R. A. , & Yates, F. (1964). Statistical tables for biological, agricultural and medical research (6th ed.). Oliver and Boyd.

[ece38417-bib-0017] Hultsch, H. , & Todt, D. (1981). Repertoire sharing and song‐post distance in nightingales (*Luscinia megarhynchos* B.). Behavioral Ecology and Sociobiology, 8(3), 183–188.

[ece38417-bib-0018] Kalko, E. K. V. (1994). Coupling of sound emission and wingbeat in naturally foraging European pipistrelle bats (Microchiroptera: Vespertilionidae). Folia Zoologica, 43(4), 363–376.

[ece38417-bib-0019] Karageorghis, C. I. , Lyne, L. P. , Bigliassi, M. , & Vuust, P. (2019). Effects of auditory rhythm on movement accuracy in dance performance. Human Movement Science, 67, 102511. 10.1016/j.humov.2019.102511 31450067

[ece38417-bib-0020] Knörnschild, M. , Feifel, M. , & Kalko, E. K. V. (2013). Mother‐offspring recognition in the bat *Carollia perspicillata* . Animal Behaviour, 86, 941–948.

[ece38417-bib-0021] Knörnschild, M. , Jung, K. , Nagy, M. , Metz, M. , & Kalko, E. K. V. (2012). Bat echolocation calls facilitate social communication. Proceedings of the Royal Society B, 279, 4827–4835. 23034703 10.1098/rspb.2012.1995 PMC3497101

[ece38417-bib-0022] Knörnschild, M. , Nagy, M. , Metz, M. , Mayer, F. , & von Helversen, O. (2012). Learned vocal group signatures in the polygynous bat *Saccopteryx bilineata* . Animal Behaviour, 84, 761–769.

[ece38417-bib-0023] Kroodsma, D. E. (2005). The singing life of birds: The art and science listening to birdsong. Houghton Mifflin Company.

[ece38417-bib-0024] Manser, M. B. (2001). The acoustic structure of suricates’ alarm calls varies with predator type and the level of response urgency. Proceedings of the Royal Society B, 268(1483), 2315–2324. 11703871 10.1098/rspb.2001.1773 PMC1088882

[ece38417-bib-0025] Marwan, N. (2008). A historical review of recurrence plots. The European Physical Journal Special Topics, 164(1), 3–12.

[ece38417-bib-0026] McRae, T. R. (2020). A review of squirrel alarm‐calling behavior: What we know and what we do not know about how predator attributes affect alarm calls. Animal Behavior and Cognition, 7(2), 168–191.

[ece38417-bib-0027] Moss, C. F. , Bohn, K. , Gilkenson, H. , & Surlykke, A. (2006). Active listening for spatial orientation in a complex auditory scene. PLoS Biology, 4(4), e79. 16509770 10.1371/journal.pbio.0040079 PMC1393756

[ece38417-bib-0028] Norton, P. , & Scharff, C. (2016). ‘Bird song metronomics’: Isochronous organization of zebra finch song rhythm. Frontiers in Neuroscience, 10(309). 10.3389/fnins.2016.00309 PMC4934119 27458334

[ece38417-bib-0029] Payne, R. S. , & McVay, S. (1971). Songs of humpback whales. Science, 173(3997), 585–597. 17833100 10.1126/science.173.3997.585

[ece38417-bib-0030] Pika, S. , Wilkinson, R. , Kendrick, K. H. , & Vernes, S. C. (2018). Taking turns: bridging the gap between human and animal communication. Proceedings of the Royal Society B, 285, 20180598. 29875303 10.1098/rspb.2018.0598 PMC6015850

[ece38417-bib-0031] Ratcliffe, J. M. , Jakobsen, L. , Kalko, E. K. V. , & Surlykke, A. (2011). Frequency alternation and an offbeat rhythm indicate foraging behavior in the echolocating bat, *Saccopteryx bilineata* . Journal of Comparative Physiology A, 197(5), 413–423. 10.1007/s00359-011-0630-0 21327333

[ece38417-bib-0032] Ravignani, A. (2018). Spontaneous rhythms in a harbor seal pup calls. BMC Research Notes, 11(3). 10.1186/s13104-017-3107-6 PMC5751680 29298731

[ece38417-bib-0033] Ravignani, A. , & Norton, P. (2017). Measuring rhythmic complexity: A primer to quantify and compare temporal structure in speech, movement and animal vocalizations. Journal of Language Evolution. 10.1093/jole/lzx002

[ece38417-bib-0034] Roalf, D. R. , Rupert, P. , Mechanic‐Hamilton, D. , Brennan, L. , Duda, J. E. , Weintraub, D. , Trojanowski, J. Q. , Wolk, D. , & Moberg, P. J. (2018). Quantitative assessment of finger tapping characteristics in mild cognitive impairment, Alzheimer’s disease, and Parkinson’s disease. Journal of Neurology, 265(6), 1365–1375. 29619565 10.1007/s00415-018-8841-8 PMC5992087

[ece38417-bib-0035] Rohatgi, V. K. , & Saleh, A. K. M. E. (2015). Statistical tables. An introduction to probability and statistics (pp. 647–665). John Wiley & Sons.

[ece38417-bib-0036] Saar, S. , & Mitra, P. P. (2008). A technique for characterizing the development of rhythms in bird song. PLoS One, 3(1), e1461. 18213370 10.1371/journal.pone.0001461 PMC2180191

[ece38417-bib-0037] Sasahara, K. , Tchernichovski, O. , Takahasi, M. , Suzuki, K. , & Okanoya, K. (2015). A rhythm landscape approach to the developmental dynamics of birdsong. Journal of the Royal Society Interface, 12(112). 10.1098/rsif.2015.0802 PMC4685852 26538559

[ece38417-bib-0038] Schnitzler, H. U. (1971). Fledermäuse im Windkanal. Zeitschrift für Vergleichende Physiologie, 73, 209–221.

[ece38417-bib-0039] Tønnesen, P. , Gero, S. , Ladegaard, M. , Johnson, M. , & Madsen, P. T. (2018). First‐year sperm whale calves echolocate and perform long, deep dives. Behavioral Ecology and Sociobiology, 72(10), 165.

[ece38417-bib-0040] Yoshida, S. , & Okanoya, K. (2005). Evolution of turn‐taking: a bio‐cognitive perspective. Cognitive Studies: Bulletin of the Japanese Cognitive Science Society, 12(3), 153–165.

