# Supplementary material for: Novel ideas to further expand the applicability of rhythm analysis
Source: Ecol Evol. 2021 Dec 6;11(24):18229–37. doi: 10.1002/ece3.8417 (PMC8717299; doi:10.1002/ece3.8417)
Supplement: Supplementary file 1 — Supplementary Material [file ECE3-11-18229-s001.docx]

# Appendix

## Modeled *ugofs* and calculated z-scores for all datasets

Here we show the modeled *ugof* values and z-scores of the best fitting beats as calculated for both methods, the IOI analysis, and Fourier analysis, corresponding to the example shown in the main manuscript for all six datasets.

We want to point out some of the similarities and differences between the datasets. In particular, the modeled *ugof* values for the three Saccopteryx bilineata datasets look very much alike, due to the fact that they all share quite similar underlying rhythms (Burchardt, Norton et al. 2019).

Z-scores vary strongly between but also within datasets, which raises some interesting questions that can well be illustrated on the sperm whale example. The dataset consists of 60 sequences, uttered by the same individual during a single dive. The individual can produce echolocation calls in a very stereotyped fashion, but it does not always do it in the same manner. An interesting question could be to correlate the z-scores to feeding success, to see if a clearer picture emerges.

Similar questions can be raised with the other datasets. It seems clear that all species are generally able to produce rhythms with a very high beat precision, i.e., very low z-scores. Nonetheless, they do not always produce them in such a fashion. The following questions hence emerge: Do we find individuals showing a higher beat precision than others? Do we find situations that show a higher beat precision than others? Can beat precision serve as a fitness signal? What are the degrees of freedom, why produce signals with a high beat precision at one point and with a lower beat precision at the next?

## Appendix Figure 1-6:

1. Distribution of ugof values of a dataset. *Ugofs* were calculated for all sequences for beat frequencies from 0.1 Hz to 100 Hz in 0.01 Hz increments, a total of 9991 *ugofs* per sequence were calculated. *Ugof* values can range from 0 to 1 as shown on the x-axis. The y-axis shows the percentage of values per bin. Number of bins: 30.
2. Z-scores are shown in dependence of the best fitting beat frequency calculated with the IOI analysis. Significant values are shown in blue, non-significant values are shown in orange.
3. Z-scores are shown in dependence of the best fitting beat frequency calculated with the Fourier analysis. Significant values are shown in blue, not significant values are shown in orange.


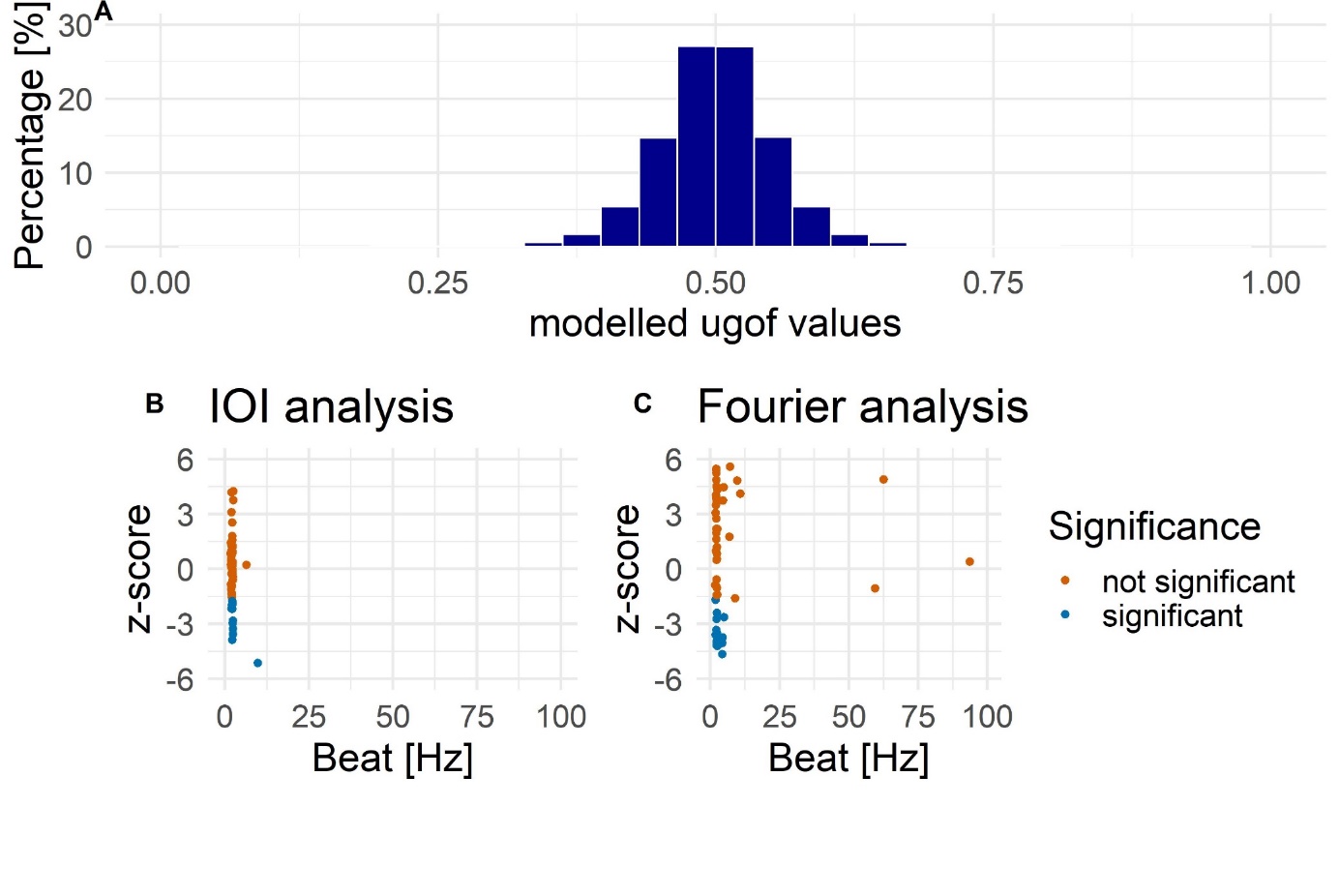


**Figure A 1: *Physeter macrocephalus*, Echolocation call sequences, n = 60, individuals = 1**


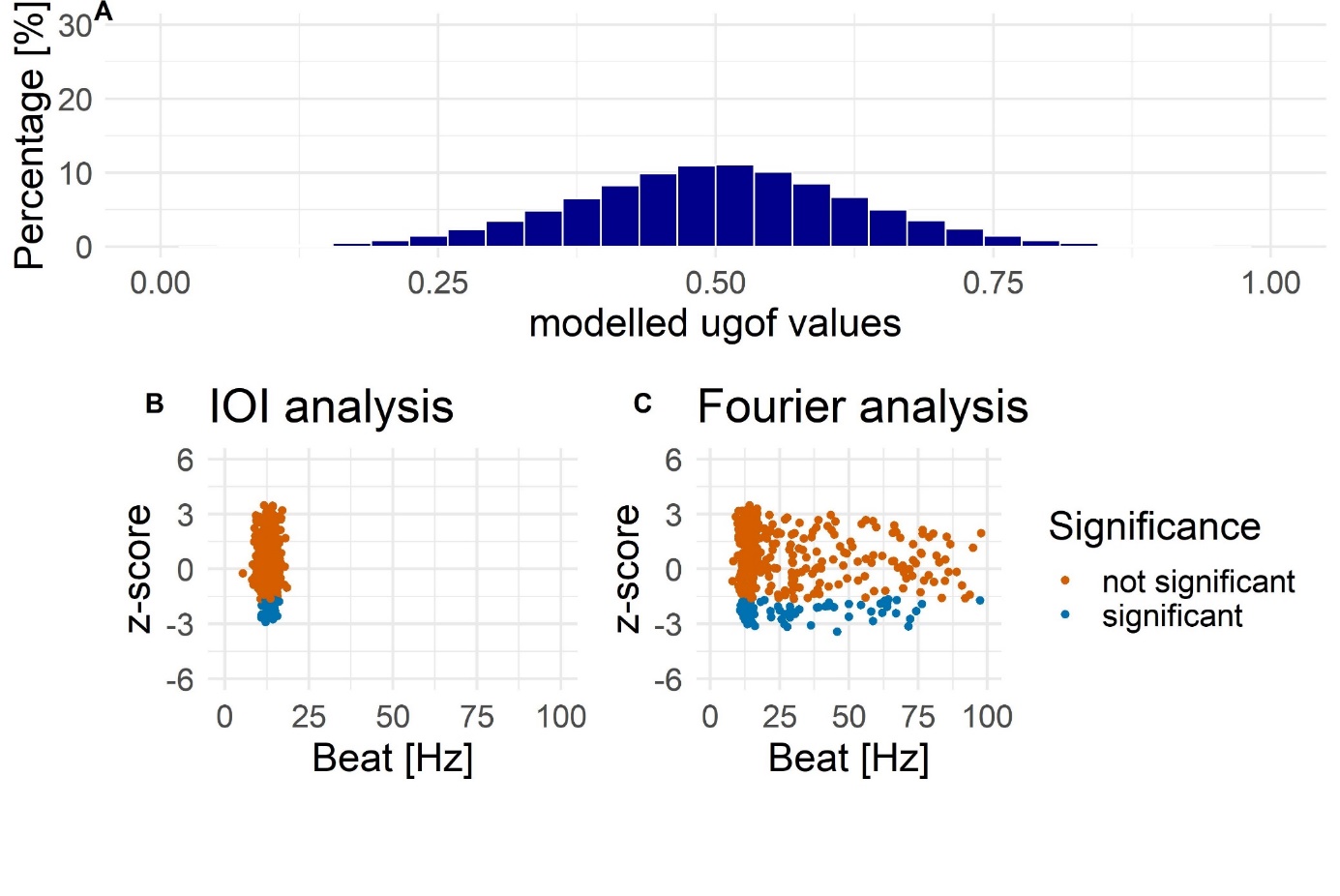


Figure A 2: *Saccopteryx bilineata*, Isolation call sequences, n = 499, individuals = 25


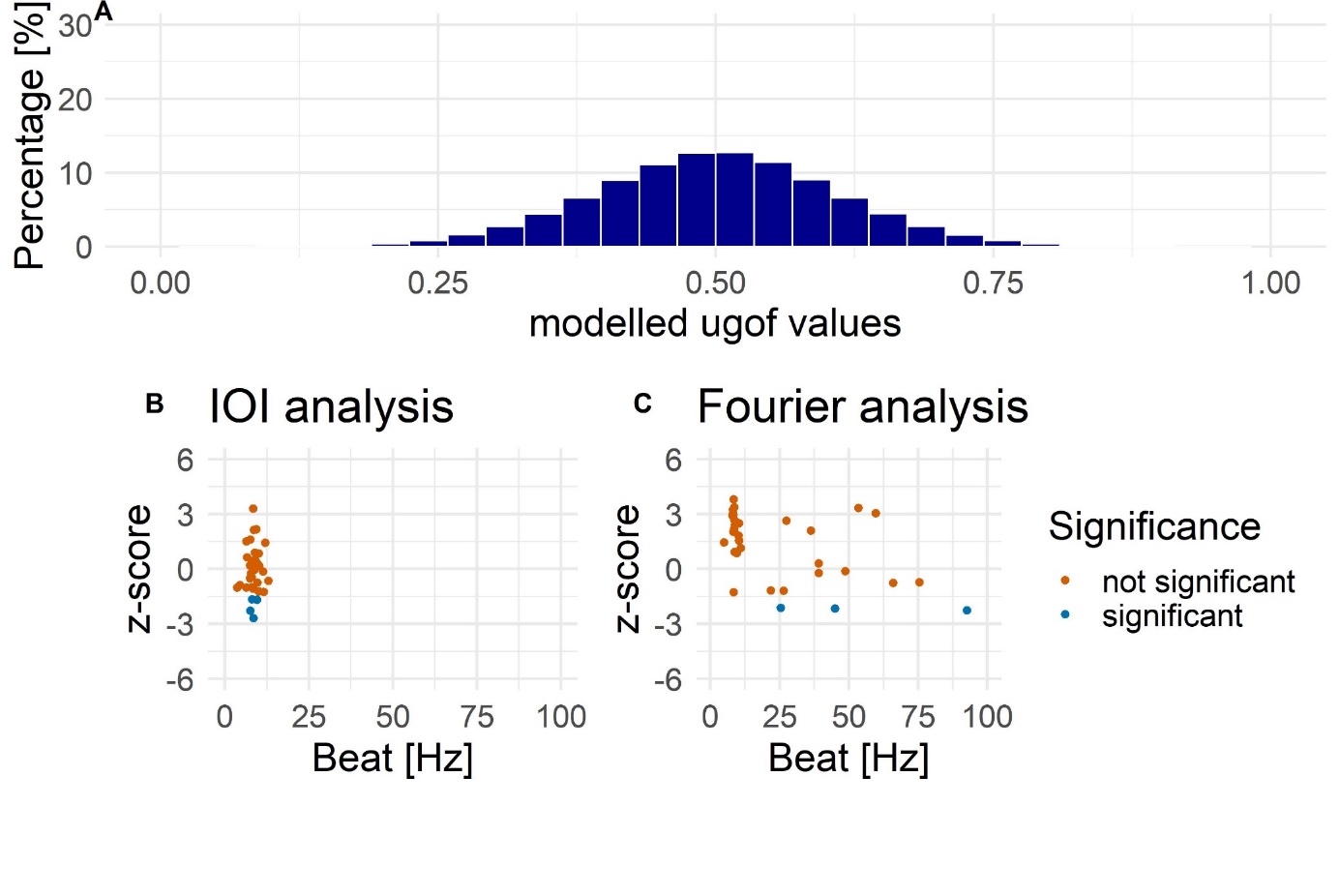


Figure A 3: *Saccopteryx bilineata*, Echolocation call sequences, n = 33, individuals = 33


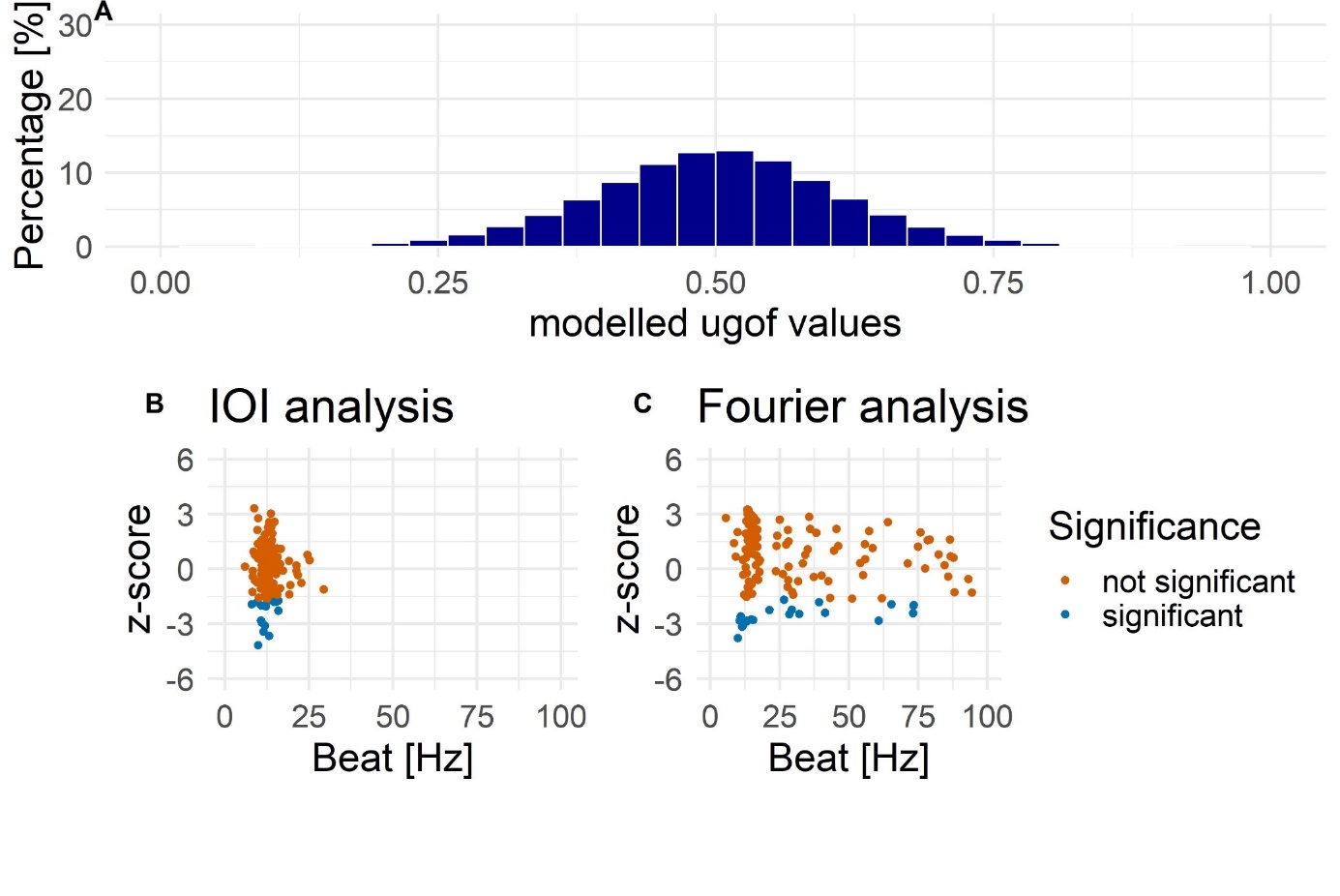


Figure A 4: *Saccopteryx bilineata*, Territorial song, n = 142, individuals = 14


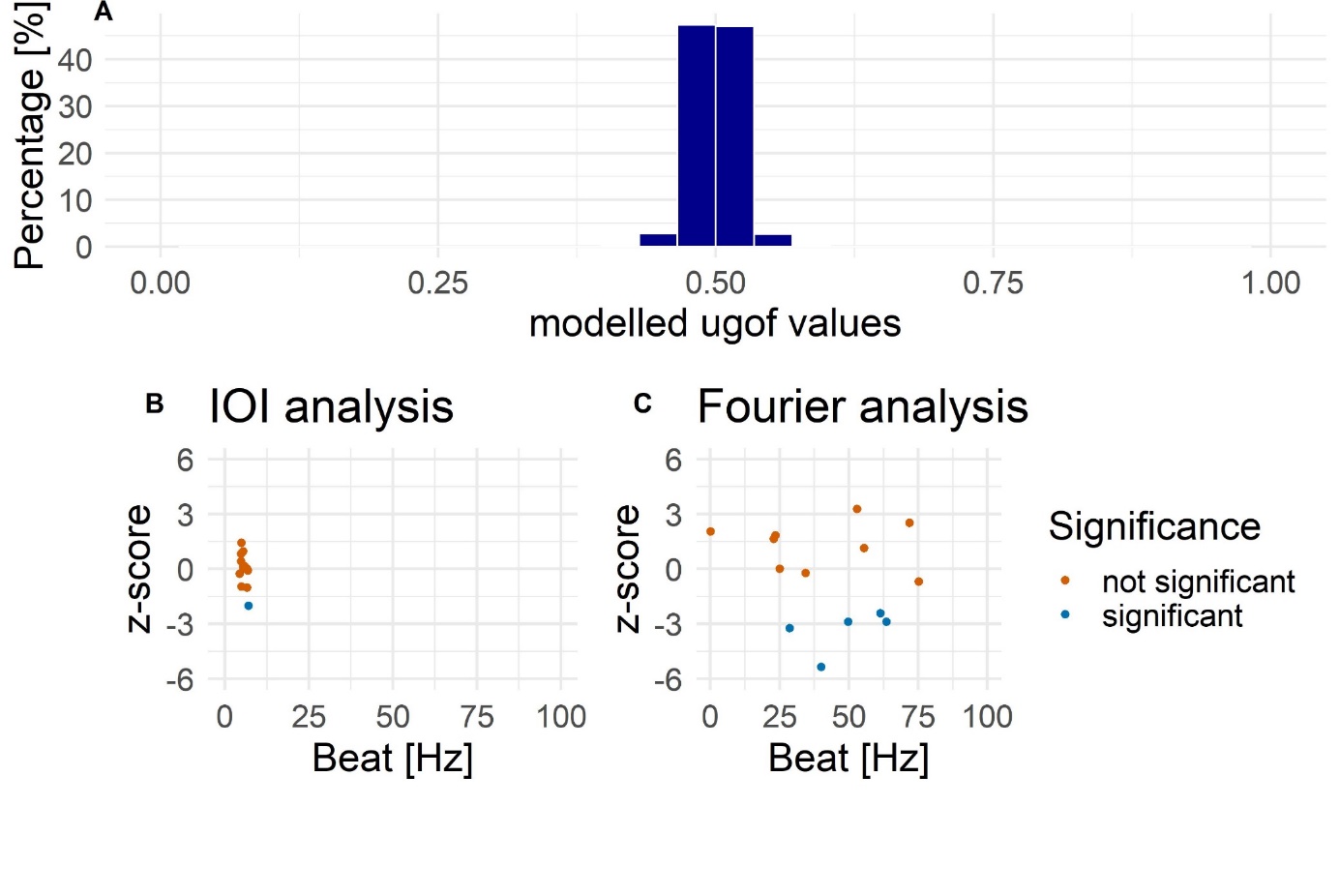


**Figure A 5: *Alauda arvensis*, Flight song, n = 14, individuals = 14**


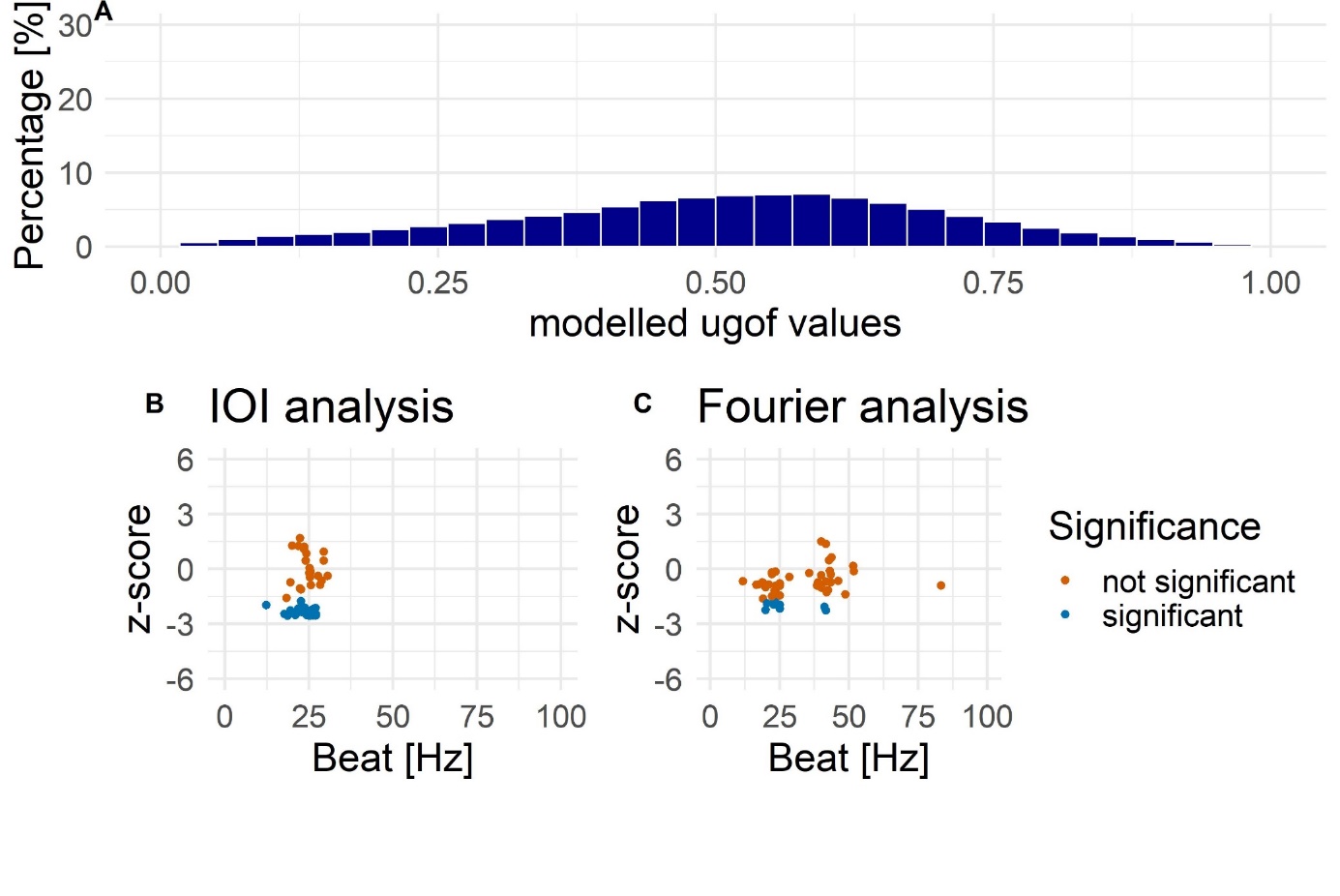


**Figure A 6: *Carollia perspicillata*, Isolation Calls, n = 49, individuals = 5**
